# Supplementary material for: Dynamic transcriptomic profiles of zebrafish gills in response to zinc supplementation
Source: BMC Genomics. 2010 Oct 11;11:553. doi: 10.1186/1471-2164-11-553 (PMC3091702; doi:10.1186/1471-2164-11-553)
Supplement: Additional file 2 — Interactive Direct Interaction Network representing the molecular interactions between zinc, copper, iron, calcium and proteins encoded by transcripts changed by zinc supplementation. Mini web-site containing index.html and hyperlinked pages in subdirectory describing a Direct Interaction Network automatically generated based on curated interactions contained within the proprietary PathwayArchitect database. Ovals represent proteins and the circles symbolize metal ions. Objects are coloured by their abundance in zebrafish at the time-point they were significantly different from the control is a scale from -4 fold (dark green) to +4 fold (dark red). Where significant differences were found at more than one time-point, the colour overlay shows expression at the first instance. Dark blue squares denote 'binding', and light blue squares 'expression'; green squares stand for 'regulation', green diamonds for 'metabolism', and green circles for 'promoter binding'. Arrow heads indicate directionality of the interaction where annotated. All nodes and edges can be further interrogated by selecting the relative area of the image. [file 1471-2164-11-553-S2.zip › PathwayArchitect Zn xs DIN/960708.html]

# REGULATION:

|  |  |
| --- | --- |
| Type | REGULATION |
| Effect | Positive |


---

|  |  |
| --- | --- |
| Score | 0 |


---

|  |  |
| --- | --- |
| Reference Count | 3 |


---

|  |  |
| --- | --- |
| Mechanism | Unknown |


---

|  |  |
| --- | --- |
| Reference:0 || Sentence | "Especially in the healthy free-living, the Zn concentration was high positively associated with the Fe concentration." |
| PMID | 11915314 |
| Year | 2002 |
| Species | Human |
| Journal | Nippon Koshu Eisei Zasshi |
| RefScore | 1 |
| Source | PArchNLP |
  |
|  |  |


---

|  |  |
| --- | --- |
 Reference:1 || Sentence | "It was shown that in addition to Ca and Fe, other minerals such as Mg, Al, and Zn accumulated in pathologic deposits." |
| PMID | 678079 |
| Year | 1978 |
| Species | Human |
| Journal | Arch Psychiatr Nervenkr |
| RefScore | 0 |
| Source | PArchNLP |
  ||  |  |


---

|  |  |
| --- | --- |
 Reference:2 || Sentence | "In vitro addition of ascorbic acid and (or) Fe identified at least two types of outliers: type 1 (approximately 70%), characterized by [Fe]Kodak > 0.4 mumol/L, by (supra)normal Fe recovery in Kodak slides in the presence or absence of ascorbic acid (10 g/L), and by between-method differences in serum Fe (Cobas - Kodak) that were significantly correlated with serum Zn content (P < 0.0004); and type 2 (approximately 30%), tentatively ascribed to contamination by EDTA, with serum Fe by Kodak < 0.4 mumol/L and Fe recovery near 0%, both of which could be significantly and dose-dependently increased by addition of ascorbic acid (5-20 g/L)." |
| PMID | 1281051 |
| Year | 1992 |
| Species | Human |
| Journal | Clin Chem |
| RefScore | 2 |
| Source | PArchNLP |
  |


---

|  |  |
| --- | --- |
